# Fostering Safety, Support, and Hope: A Qualitative Study of Multistakeholder Perspectives on Designing Health Care Services for Women Who Use Drugs in Seattle, Washington, 2023

**DOI:** 10.1177/26884844251372851

**Published:** 2025-08-27

**Authors:** Elizabeth J. Austin, Jenell Stewart, Lauren R. Violette, Sara N. Glick, Shireesha Dhanireddy, Emily C. Williams, Maria A. Corcorran

**Affiliations:** ^1^ Department of Health Systems and Population Health, University of Washington, Seattle, Washington, USA.; ^2^ Division of Infectious Diseases, Hennepin Healthcare Research Institute, Minneapolis, Minnesota, USA.; ^3^ Department of Medicine, University of Minnesota, Minneapolis, Minnesota, USA.; ^4^ Division of allergy & Infectious Disease, Department of Medicine, University of Washington, Seattle, Washington, USA.; ^5^ Department of Population Medicine, Harvard Medical School, Boston, Massachusetts, USA.; ^6^ Harvard Pilgrim Health Care Institute, Boston, Massachusetts, USA.

**Keywords:** gender-based care, harm reduction, drug use, social determinants

## Abstract

**Background:**

Women who use drugs (WWUD) experience increasingly worse outcomes from drug use as compared to men. Additionally, transactional sex, unstable housing, and unmet needs may further complicate their ability to get needed health care. To inform the design of gender-based, mobile health services, we sought perspectives on health care service delivery from WWUD and health care and harm reduction professionals (HHRPs) in Seattle, WA.

**Materials and Methods:**

In 2023, we conducted qualitative interviews with WWUD (n = 16) and HHRPs (n = 5) in the Seattle, Washington area. Interviews focused on experiences receiving health care services, barriers and facilitators to accessing care, and preferences for health care service delivery. Interviews were audio recorded, transcribed, and double coded. Final learnings were reviewed with the study’s community advisory boards.

**Results:**

All WWUD participants identified as cisgender women, 12 (75%) identified as White, and 11 (69%) had unstable housing. Analysis identified four themes that characterized perspectives of WWUD and HHRP on health care service delivery needs, embodied by: safety, stigma, hope, and resiliency. Participants described that lack of safety and unmet basic needs made it challenging for WWUD to ever feel healthy (theme 1), and that WWUD felt stigmatized by mainstream health care services in intersectional ways (theme 2). However, participants shared that these burdens were lessened when health care teams created space for hope in care delivery interactions (theme 3) and built resiliency by reducing barriers and complementing care with structural supports (theme 4).

**Discussion:**

In this small qualitative sample, WWUD experienced a myriad of intersecting challenges that perpetuated marginalization and health disparities. This population may benefit from interventions rooted in intersectional and trauma-informed approaches.

## Introduction

Women who use drugs (WWUD) experience disproportionate harms from substance use, when compared to the population of people who use drugs as a whole.^1–3^ Over the past few decades, rates of drug use, drug overdose, and drug-related criminal offenses have continued to rise among women and in some cases disproportionately so; for example, incarceration for drug-related offenses has increased by 800% for women in the past few decades, as compared to a 300% increase for men.^4–6^ While there are persistent efforts to scale-up harm reduction service delivery, these services are often male-dominated and fail to engage and create a safe space for WWUD.^7^ As a result, WWUD are less likely than men to use syringe services programs and safe consumption sites, be on HIV pre-exposure prophylaxis, be treated for Hepatitis C virus (HCV), or receive other sexually transmitted infection (STI)-related services, and less likely to start and stay in substance use treatment.^8–11^ It is therefore imperative to understand how to address and reverse the rising disparities in health outcomes for WWUD.

WWUD also experience multiple, intersecting forms of stigma and discrimination that directly contribute to decreased social supports and capital, increased risk behaviors, and experiences of gender-based violence.^2,4,5^ For example, a 2020 cross-sectional study in Kazakhstan found that among WWUD who also engage in transactional sex, 90% had experienced lifetime violence, including physical violence (88%), sexual violence (79%) or psychological violence (72%), and half experienced violence in the past 90 days, primarily from transactional sex clients or police.^12^ Multiple studies in the United States also show that WWUD who engage in transactional sex are more likely to experience homelessness, economic deprivation, condomless sex, and harms from drug use.^13–15^ One study specific to the Seattle area—the setting of the present study—found that of 144 WWUD who also inject drugs, 70% had an income level below the poverty line and 76% had experienced homelessness in the past year.^14^ The impact of these intersecting risks for WWUD may vary regionally and that variation is underrepresented in the literature. More importantly, these intersecting risks are under-addressed in the approach to health care service delivery for WWUD.^5,12,16,17^ This is a critical gap that will continue to perpetuate harms for WWUD.

Prior research on the experiences of WWUD repeatedly echo the importance of designing health care services around the unique preferences and needs of women, in particular considerations for safety, trauma, and intersecting risks (*i.e.,* syndemics).^5,16^ Yet few articles have explored the perspectives of WWUD on specific preferences and recommendations to tailor health care services to enhance engagement. In 2018, a team of clinician researchers launched a low barrier, women-focused clinic in a Seattle-based community setting that demonstrated success at reaching WWUD who might otherwise not be able to access or engage in mainstream health care services. The clinic addressed key clinical needs for WWUD including substance use treatment (*e.g.,* medications for opioid use disorder), HIV/STI testing and prevention services, and STI treatment.^18–20^ Despite its success at delivering accessible, gender-based services to WWUD, many WWUD in the community still had unmet needs. As part of a formative evaluation to codesign the expansion of these services, particularly for WWUD who are unable to access the low barrier clinic, we sought to characterize the perspectives of WWUD and the health care and harm reduction professionals (HHRPs) who provide services for them on health care delivery experiences, preferences, and needs.

## Materials and Methods

We conducted qualitative interviews with a convenience sample of WWUD and HHRPs in Seattle, Washington. WWUD were eligible if they were 18 years of age or older, spoke English, identified as a woman regardless of the sex they were assigned at birth, and reported a lifetime history of injection drug use. HHRP participants were eligible if they worked closely with WWUD in care settings (inpatient or outpatient), shelters, or community-based harm reduction service settings. Recruitment occurred between March and May of 2023 through word of mouth and in-person referrals from three community-based organizations that provide harm reduction services to WWUD. We also used passive recruitment strategies of posting flyers in clinic and community spaces where WWUD may frequent. Eligible and interested participants were approached by a member of the study team and provided verbal informed consent before participating in one semistructured interview. At multiple points during recruitment, the study team met to discuss the representativeness and saturation of sample and make needed adjustments to recruitment efforts. We aimed to recruit 15 WWUD and we ultimately screened 19 WWUD and enrolled 16 WWUD in the study. We aimed to recruit five HHRPs, and all five HHRP who were screened were eligible and participated in the study.

Interviews were conducted by three interviewers (L.R.V., M.A.C., and J.S.) who identify as cisgender women and have experience working with WWUD. Interviews with WWUD were conducted at locations where participants already received harm reduction services to increase trust and safety during data collection, including a day drop-in center, a syringe services program, and a mobile methadone program.^21^ Interviews with HHRPs were conducted virtually. Participants (WWUD and HHRP) received $50 USD for interview completion. Interviews followed a semistructured discussion guide developed by the full study team, including researchers with expertise in substance use and infectious disease care, health services research, and implementation research. The discussion guide was also reviewed by the study’s community advisory boards, including people with lived experience receiving or delivering harm reduction services. Interviews asked open-ended questions about perceptions of health and wellbeing (WWUD), experiences receiving health care (WWUD) or delivering health care (HHRP), and preferences for health care delivery that would be more responsive to needs (WWUD and HHRP). WWUD participants also completed a brief survey *via* REDCap that asked about demographics and prior patterns of drug use.^22^ On an average, interviews lasted between 20 and 50 minutes for WWUD and between 30 and 60 minutes for HHRP. All participants received $50 for participating in the study.

Interviews were audio-recorded, professionally transcribed, and coded using Rapid Assessment Process (RAP) by a team of qualitative coders (E.J.A., M.A.C., and L.R.V.). RAP is a team-based analysis method that combines deductive and inductive approaches to data reduction and iterative theme development.^23–25^ Coding unfolded in four iterative rounds. For each round, two coders (*i.e.,* double coding) generated templated coding summaries for each interview transcript utilizing a coding schema informed by interview domains. Double-coding was compared for consistency and aligned *via* consensus. Initial coding was done for WWUD and HHRP participants separately, so as to gain a preliminary understanding of their unique perspectives. Then, coding summaries for all participants were combined and placed into a matrix in Microsoft Excel and reviewed iteratively to identify cross-cutting themes. Emerging data interpretations were documented *via* qualitative memos and aligned with existing social epidemiological theory related to harm reduction, intersectionality, and risk environments.^16,26,27^ Analysis also compared and contrasted perspectives of WWUD and HHRP participants to generate a more comprehensive integration of perspectives on care delivery experiences. Qualitative findings were reviewed with the investigative team and confirmed with the study’s community advisory boards. All activities were reviewed and approved by the University of Washington Institutional Review Board.

## Results

Participants in our sample included 16 WWUD and 5 HHRPs. Among WWUD participants, most (n = 12, 75%) identified as White and most (n = 11, 69%) were experiencing unstable housing. All WWUD had lifetime drug use experience and almost half (n = 7, 44%) had injected drugs in the past 6 months, with the most common drug injected being methamphetamine (n = 13, 81%) followed by heroin and methamphetamine combinations (n = 9, 56%) or heroin alone (n = 7, 44%). While all WWUD participants had a lifetime history of injection drug use, many were using substances *via* other routes at the time of the interview, so we use the term WWUD to be more inclusive of their experience. Many WWUD participants also described current or past experiences with transactional sex. Table 1 offers more details about the sample of WWUD. HHRP participants mostly identified as cisgender women and White and many had 10 years or more of experience working in HIV and substance use care delivery (see Table 2). Analysis of qualitative data identified four themes, detailed in the following section.

**Table 1.** Demographic Characteristics of WWUD Interviewed (*n* = 16)

Demographic characteristic	*n* (%)
Age	
≤20	0 (0%)
21–30	3 (19%)
31–40	4 (25%)
41–50	5 (31%)
51–60	3 (19%)
61+	1 (6%)
Current gender identity	
Cisgender woman	16 (100%)
Racial/ethnic identity^a^	
American Indian/Alaska Native	4 (25%)
Black or African American	4 (25%)
Hispanic/Latina	1 (6%)
Asian	1 (6%)
White	12 (75%)
Other (“Mixed”)^b^	1 (6%)
Missing	1 (6%)
Current living situation	
Couch surfing (not on a lease)	3 (19%)
Own home or apartment	5 (31%)
On the street or in a vehicle	3 (19%)
Overnight shelter	1 (6%)
Transitioning housing	4 (25%)
Injected any drug in the past 6 months?	
Yes	7 (44%)
No	8 (50%)
Don’t know	1 (6%)
Drugs ever injected (check all that apply)^c^	
Methamphetamine by itself	13 (81%)
Goofballs (heroin and methamphetamine)	9 (56%)
Heroin by itself	7 (44%)
Speedballs (heroin and cocaine)	5 (31%)
Fentanyl with another drug	4 (25%)
Fentanyl by itself	3 (19%)
Powder cocaine	3 (19%)
Crack cocaine	4 (25%)
Painkillers	1 (6%)
Phenobarbital	1 (6%)
HRT	1 (6%)
Benzodiazepines	0 (0%)
Xylazine/tranq	0 (0%)
Ketamine	0 (0%)
Buprenorphine or suboxone by itself	0 (0%)

^a^ Responses are not mutually exclusive.

^b^ One participant wrote in “mixed” for racial identity.

^c^ One survey was asked about past 6-month injection before question was changed to lifetime. Reported meth and goofball use.

WWUD, women who use drugs.

**Table 2.** Demographic Characteristics of HHRP Interviewed (*n* = 5)

Demographic characteristic	*n* (%)
Current gender identity	
Cisgender women	3 (60%)
Cisgender men	1 (20%)
Nonbinary	1 (20%)
Racial/ethnic identity	
American Indian/Alaska Native	0 (0%)
Black or African American	0 (0%)
Hispanic/Latina	0 (0%)
Asian	1 (20%)
White	4 (80%)
Current professional title	
Physician	3 (60%)
Patient advocate	1 (20%)
Executive director	1 (20%)
Years worked in HIV-related services	
0–5	1 (20%)
6–10	1 (20%)
11–15	2 (40%)
16+	1 (20%)
Years worked in substance use-related services	
0–5	1 (20%)
6–10	1 (20%)
11–15	2 (40%)
16+	1 (20%)

HHRP, harm reduction professionals.

### Theme 1: Lack of safety and unmet basic needs make it challenging for WWUD to feel healthy

When WWUD talked about their health, they shared a holistic view on health and wellbeing. WWUD described that being healthy involved a combination of healthy body and mind, being able to care for oneself, and engaging in health-promoting habits. As these participants shared:

“Healthy means a positive mind, a positive, your body feels good, you feel energized, you feel good. Your emotional, physical, mental… everything has to be squared away.” [P16, WWUD]

“Being healthy means eating right, sleeping right, being able to do just things I need to do during the day and not feel lethargic.” [P12, WWUD]

However, WWUD shared that achieving that state of holistic health felt difficult, as trying to meet basic needs often took precedence in their daily lives. One participant described this, saying that their focus was on “*just being able to survive on a daily basis […] without being worried about it all the time*” [P5, WWUD]. In particular, WWUD expressed that concerns about their safety permeated their daily experience. Women described being exposed to violence and harms regularly, including among those in their immediate circle of relationships. As one participant shared:

“Everybody has been dying, right? Two really good friends had died on the same day the other day and then my best friend got murdered last month—or two months ago. It's dangerous down here. It's a risk coming out here and—working [city neighborhood] is an extreme risk and it's getting worse. I don't know why exactly. Yeah, it's dangerous out here” [P1, WWUD]

WWUD shared that they thought about their safety “*a lot, quite a bit*” [P12, WWUD] or “*almost every day, every minute*” [P3, WWUD]. This included thinking about the way they presented themselves when they went out in public.

“Especially if I leave the house looking nice. […] I made a point of, at the time I lived there, to never leave the house with makeup on. Never wear a dress. Always be bundled up. Cover my whole face if possible. Don’t swish my hips when I walk. Just stay as sexually benign as possible so that you don’t call attention to yourself ‘cause there were some aggressive men in that neighborhood.” [P4, WWUD]

Another participant described thinking about safety in terms of the times they went outside and places they went, and the fear of being more likely to be kidnapped or harmed at certain times of day.

“And more of the safety part as schedule wise, like being out here–some people get took and get hidden and stuff like that. So, that’s what I’m more concerned about right now.” [P6, WWUD]

HHRP also echoed the constant threat of violence many WWUD faced. As one HHRP shared:

“I think when I’m addressing the people who exchange sex, or at least who talk about it more, it’s really an additional focus on staying on physical safety. And because we know that these women are at risk […] Risk of assault; risk of sexual assault, physical assault; so—yeah. Risk of murder. There’s a lot of violence.” [P8, HHRP]

Participants shared that this constant focus on safety and survival made it hard for WWUD to ever feel a true sense of wellbeing. As one WWUD summarized:

“I want to be able to go home and be able to sleep knowing that I’m going to wake up okay the next morning and not wake up dead.” [P3, WWUD]

### Theme 2: WWUD feel stigmatized and shunned by mainstream health care in intersectional ways

WWUD described prior experiences seeking health care services that made them feel unworthy and unwanted. WWUD felt that health care teams “*look down on them and they shun them*” [P18, WWUD] or that “*they’re just rude*” [P16, WWUD]. Participants shared that they felt stigmatized by health care teams for multiple reasons, including health care team perceptions that participants were seeking drugs, exchanged sex, or were unstably housed, regardless of whether these things were true. As these participants shared:

“They’re being shame-on-you kind of attitude. And ‘Oh I don’t want to help you because you’re homeless or because you look dirty or because you carry a wheeling cart.” [P3, WWUD]

“Once again, when people aren’t empathetic, when they don’t think you’re worth the healthcare. At least you feel that way. I don’t know if they mean to make you feel that way, but they make you feel that way, like you don’t matter in the world.” [P5, WWUD]

“But then when you come to a point where you need pain meds, and you go to the doctor, and you tell the doctor, ‘Well, this is what I’ve been given in the past. This is what works for me.’ ‘Oh, you’re a drug seeker. You’re a pill popper.’ Okay, how did you get that from our conversation? I don’t understand that. […] You don’t even know me, and you already assume I’m here just to seek drugs.” [P3, WWUD]

HHRP echoed the role of stigma in health care seeking experiences of WWUD, saying:

“We are taught by other people that we are worthless. Especially as women, especially when you are out on [street name], you are shown and taught every single day that you don’t matter. And so, it’s impossible not to internalize that.” [P14, HHRP]

Disclosing past or current drug use felt like a specific form of entrapment to WWUD that often led participants to receive inadequate care and increased discrimination from health care staff. Several participants described experiences where they felt health care teams did not take their medical concerns seriously or did not provide appropriate medical care because of drug use stigma:

“And they were so mean. They were cool until they asked me if she did drugs. And we were always told be honest. And so, every time they ask us that question and it’s not in this room here, we know we’re taking a risk of this now being turned into, ‘Well, it’s your addiction. You’re dope sick.’” [P1, WWUD]

“I got leg pain, pains all around. Won’t help me with that. They won’t, because I’m a drug addict, they won’t administrate some medicines and stuff like that to a lot of people. Because they’re quick to judge. They’re quick to look down on people.” [P18, WWUD]

As a result, WWUD felt a sense of hopelessness about seeking care for their health care needs and often delayed care as a result.

“I would put off going to the hospital as much as possible—as long as possible—because of the treatment that they would give us when we get there. And so, that would lead us to basically doing things like popping our own abscesses, which is completely a horrible situation and causes more infection.” [P1, WWUD]

“And so, that really defeats the purpose of somebody seeking help. And then less and less people want to go seek help because they’re being judged.” [P3, WWUD]

Ultimately WWUD believed that reducing the stigma they felt when seeking health care services would play an important role in supporting their health.

### Theme 3: Combating stigma by creating space for hope in patient-provider interactions

Both WWUD and HHRP emphasized the detrimental role stigma played in the health of WWUD. In response to this, HHRP described efforts to counteract the impacts of stigma through kindness and *“creating a space where they do matter”* [P14, HHRP] in their interactions with the WWUD they served. One HHRP shared that *“we put kindness and saying ‘yes’ kind of at the forefront”* [P8, HHRP]. Another described their goal to help WWUD feel safe and supported, saying:

“I think a lot of the times when people have that space to just let their guard down and actually dream, if that makes sense. It’s kind of cheesy, but if that makes sense, that is where change happens. That’s how people are able to make changes, not when they’re forced.” [P14, HHRP]

WWUD who received services from settings that focused on harm reduction and gender-based services acknowledged the difference in how service delivery felt to them. One WWUD shared the importance of *“just people having your back and helping to support you, to help you to get there, to be someone to talk to. […] They understand that I’m just trying to do the best I can do, and I think that they respect that”* [P11, WWUD]. Yet another expressed the importance of getting services in settings where they felt valued as a person:

“Because when I go in there I tell them what I’m feeling, what I need, what I expect, and they treat me like a person. They treat me with love and care like they should.” [P3, WWUD]

WWUD and HHRP participants shared that a key difference in these services was the space that harm reduction settings created for WWUD to express needs, feel valued, and foster hope for improving their health and wellbeing. One HHRP summarized this, saying:

“It’s creating space, literally creating space to just allow the women on [street name] to exist, to feel, to cry, to laugh, to do literally whatever they need to feel human. That’s what I feel is at the core of it. […] I think just trying to be—I don’t know, someone with a headlamp on in the really dark system like the tunnels of the really broken systems of homeless services, mental health services, substance use services, healthcare, all the things.” [P14, HHRP]

### Theme 4: Building resiliency by reducing barriers and increasing structural supports

Participants described that WWUD often face many structural barriers to accessing the care they need. Lack of stable housing was one such example of a structural barrier to accessing care, maintaining a schedule that supported care adherence, or even contributed to the stigma WWUD felt by health care teams:

“Just being on the streets, not keeping track of the days or the hours and living a different life that was unstable and made it harder to go to doctor’s appointments and make sure I was there when I was supposed to be” [P11, WWUD]

“Yeah, because they don’t think you’re going to pay your bills, or you don’t have a place to mail your bill to or anything like that. So, that makes it a big issue for a lot of places.” [P5, WWUD]

Other participants shared examples that ranged from limited phone access to unreliable public transportation or the threat of police presence, highlighting the ways structural barriers influence many aspects of daily life for WWUD. As one HHRP described:

“And especially because they often don’t have phones, they don’t have—they’re doing street-based sex work, so they don’t have a home base, necessarily, they’re very vulnerable to abusive customers. And highly judged by the safety nets of ER and police, who are supposed to be there to help take care of them, as well.” [P8, HHRP]

Participants shared that while health care services could not solve many of these structural barriers, there were opportunities to make care easier for WWUD to utilize. WWUD participants expressed that “*it would be nice to have evenings and weekends available*” [P12, WWUD], as well as opportunities to combine services in a single appointment. HHRP participants echoed the importance of increasing accessibility, both in terms of schedules and ease of access, saying:

“Accessible; good hours; good days; we don’t need a Monday morning from 7:00 a.m. to noon clinic. […] I think the women who are exchanging sex are usually working in the evenings, but sometimes in the mornings they are done for the day, they’re gonna have a meal, maybe go get some sleep, maybe go get a shower. I think what has really helped our clinic is having connection to a larger health infrastructure, so that if imaging studies are needed; if more than what I can do in the clinic is needed, then women can access that without going through the emergency department.” [P8, HHRP]

HHRP participants emphasized the importance of designing care around the needs of patients, not the system. HHRPs described this as being *“ready to strike when the opportunity presents itself” [P19, HHRP]*, and shared their goal of providing services that never created more barriers for WWUD, saying:

“I would say my stated goal is like to try to say “Yes” as much as possible. That everywhere people go, people are saying, “No, not right now.” I think that that—I try to say “Yes” and try to figure out ways that we can get to yes, if there’s limitations that I have in the care that I’m providing where I can’t say “Yes,” to try to come up with something a little close—closer to that.” [P8, HHRP]

This low-barrier approach meant looking for opportunities to provide maximal flexibility, centering treatment delivery around the things individual patients felt able to do at each visit. For example, one HHRP described offering to hold medications onsite to help WWUD feel able to maintain their supply and access it when needed:

“That requires a proactive and outreach-based approach whereby we really just say, you know, I would really like to order this medication for you in case and we will hold onto it for you in case. It’s still your medicine. You can take the entire fill of Suboxone with you, if you wish, to keep with you on your person, but we’d be happy to hold onto it for you, as well. And so, kind of just really offer these low-barrier opportunistic treatment options, and really, also offer a menu of options. I think choice is extremely important for people.” [P19, HHRP]

WWUD participants who had received care that aimed to reduce barriers instead of contribute to them expressed the value of this approach:

“Well, they [community service setting] saw that I needed healthcare, so they got me healthcare. They just did it. They just figured out what I need. And they don’t force me to do the things, but they suggest it, and then, if it’s something that I want to do, then they’ll take care of it for me usually or provide ways to get me there, so that it can get done” [P13, WWUD]

## Discussion

This work explored the perspectives of WWUD and HHRP on health care experiences, preferences, and needs for WWUD, including the intersectional experiences of drug use, transactional sex, housing instability, and gender-based violence. The themes that emerged from participants characterized health care experiences fraught with negativity and unmet needs, as well as persistent structural barriers to achieving health for WWUD. However, participants also described promising approaches to tailor care delivery—both structurally and individually—in ways that acknowledge and actively reduce the burdens WWUD face in their daily lives.

Learnings from this study demonstrated that WWUD do not make decisions about health in a vacuum—instead WWUD are strongly influenced by stigma, external stressors, and the risk environments in which they live.^17^ Risk environments involve physical, social, economic, and policy factors that individually and collectively increase risk for substance use, HIV, and harms across all aspects of health and wellbeing for WWUD and exchange sex.^16,27,28^ For the participants in our study, safety risk of environments directly influenced the health of WWUD. Lack of safety, for example, permeated all aspects of WWUD daily life including limiting their interactions with their physical environment, their ability to access economic resources, and ability to achieve social and emotional wellbeing. So, while increasing the accessibility of health care services, such as through expanded locations and hours of operation, is important for WWUD, it will likely be more impactful if paired with strategies that increase perceived and actual safety for WWUD when seeking those services.

This work contributes to understanding the multidimensionality of lived experiences for WWUD and how those can inform needed multilevel and structural interventions that begin reversing the negative impacts of risk environments.^5,16,28^ As was echoed in our findings, existing policies surrounding criminalization of drug use and sex work-when paired with the undercurrent of societal expectations for women and gender-based violence—differentially impact women and limit their ability to access needed health care and social supports, while simultaneously reinforcing male power and control. In contrast, changes in national or local policies that decriminalize sex work and reduce gender-based violence would work in concert to increase women’s access to autonomy, resources, and safety.^4,28,29^

These data also reinforce prior work characterizing the pervasive presence of stigma in health care experiences for WWUD, often leading WWUD to feel unworthy of seeking health and wellbeing.^2,4,5^ Stigma serves a historical, societal, and political purpose to maintain hierarchy through othering and shaming.^30^ Participants in our study shared examples of experiencing expressed, anticipated, and internalized stigma related to multiple, intersecting aspects of their identity.^31,32^ Their examples illuminated how stigma infiltrated their lived experiences and negatively influenced activities of health promotion, health seeking, and risk, thus perpetuating cycles of marginalization. Recognizing the ways that WWUD have been harmed by intersectional stigma must be at the forefront of how we respond with clinical and public health interventions; health care delivery settings should anticipate and address stigma *via* practice culture and messaging for staff, providers and patients. Too often, stigma interventions fail to acknowledge the intersectionality women experience and focus exclusively on the stigma associated with drug use generally.^4,32^ Instead, health care settings should consider the ways that WWUD may feel overlapping forms of stigma related to drug use, drug use and sex work, drug use and intimate partner relationships and often violence, and drug use and motherhood. This could result in approaches to patient education and care delivery that better address the unique challenges WWUD may face, for example, accessing childcare or negotiating safer drug use practices.^33^

Finally, our participants shared the importance of care delivery approaches centered in trauma-informed care and focused on building resiliency and fostering hope. For WWUD, this may include intentional strategies to help women feel safer during health care interactions and more able to disclose and discuss trauma that impacts their health and wellbeing.^34,35^ For example, women seeking care may need additional assurances that care delivery settings are private and confidential. Explicit discussions about privacy and confidentiality between patients and providers may illuminate additional needs, such as preferences for health care provider gender and/or lived experience, preferences for the physical layout of exam rooms (*e.g.,* doors open or closed, options for women to feel able to leave settings if they feel unsafe), and preferences for what aspects of their history and care they are ready to discuss. These intentional actions are examples of way to acknowledge that women may experience limited control or power in other aspects of their life, but have an opportunity to change that dynamic in their health care interactions. Using a trauma-informed, strengths-based approach will be essential, not only for helping individual women heal from the ways intersectional stigma has harmed them, but also for rebuilding trust that has been lost between communities of WWUD and care delivery settings.^12^

There are several limitations to acknowledge in this study. Our sample reflects a convenience sample of WWUD and HHRPs that is limited in its racial and ethnic diversity and, though not by design, did not include transgender individuals. Future research should continue to explore more diverse perspectives and intersectional experiences of WWUD. Additionally, our sample was recruited from a single geographic region with unique structural factors (*e.g.,* local policies, community resources) that may not be transferable to other regions. Finally, qualitative data are intended to provide in-depth understandings of lived experience; the learnings from this study are not intended to be generalizable and should be complemented by additional research that expands the samples and methodological approaches used.

## Conclusions

Findings from this qualitative study suggest that WWUD experience a myriad of intersecting challenges that influence their health care experiences and perpetuate health disparities. Utilizing an intersectional and resiliency-focused approach to understand lived experience, particularly the ways in which substance use, transactional sex, housing instability, and gender-based violence overlap, will improve the responsiveness of public health interventions that aim to address the needs of WWUD and improve linkages to care.

## Data Availability

Data from this study will be made available upon reasonable request to the corresponding author.

## Abbreviations Used

HCV: Hepatitis C virus
HHRP: health care and harm reduction professionals
RAP: rapid assessment process
STI: sexually transmitted infection
WWUD: women who use drugs
